# Immunopathogenesis and treatment of cytokine storm in COVID-19

**DOI:** 10.7150/thno.49713

**Published:** 2021-01-01

**Authors:** Jae Seok Kim, Jun Young Lee, Jae Won Yang, Keum Hwa Lee, Maria Effenberger, Wladimir Szpirt, Andreas Kronbichler, Jae Il Shin

**Affiliations:** 1 Department of Nephrology, Yonsei University Wonju College of Medicine, Wonju, Republic of Korea.; 2 Department of Pediatrics, Yonsei University College of Medicine, Seoul, Republic of Korea.; 3 Department of Internal Medicine I, Gastroenterology, Hepatology, Endocrinology & Metabolism, Medical University Innsbruck, Innsbruck, Austria.; 4 Department of Nephrology, Rigshospitalet, University of Copenhagen, Copenhagen, Denmark.; 5 Department of Internal Medicine IV, Nephrology and Hypertension, Medical University Innsbruck, Innsbruck, Austria.

**Keywords:** Coronavirus, COVID-19, cytokine storm, cytokine blockades, plasma exchange

## Abstract

Severe coronavirus disease 2019 (COVID-19) is characterized by systemic hyper-inflammation, acute respiratory distress syndrome, and multiple organ failure. Cytokine storm refers to a set of clinical conditions caused by excessive immune reactions and has been recognized as a leading cause of severe COVID-19. While comparisons have been made between COVID-19 cytokine storm and other kinds of cytokine storm such as hemophagocytic lymphohistiocytosis and cytokine release syndrome, the pathogenesis of cytokine storm has not been clearly elucidated yet. Recent studies have shown that impaired response of type-1 IFNs in early stage of COVID-19 infection played a major role in the development of cytokine storm, and various cytokines such as IL-6 and IL-1 were involved in severe COVID-19. Furthermore, many clinical evidences have indicated the importance of anti-inflammatory therapy in severe COVID-19. Several approaches are currently being used to treat the observed cytokine storm associated with COVID-19, and expectations are especially high for new cytokine-targeted therapies, such as tocilizumab, anakinra, and baricitinib. Although a number of studies have been conducted on anti-inflammatory treatments for severe COVID-19, no specific recommendations have been made on which drugs should be used for which patients and when. In this review, we provide an overview of cytokine storm in COVID-19 and treatments currently being used to address it. In addition, we discuss the potential therapeutic role of extracorporeal cytokine removal to treat the cytokine storm associated with COVID-19.

## Introduction

Coronavirus disease 2019 (COVID-19) has affected the world in a devastating way since December 2019. The causative virus, severe acute respiratory syndrome coronavirus 2 (SARS-CoV-2), has gained novel properties through variations in its original form. It now has great infectivity and is often fatal, causing acute respiratory distress syndrome (ARDS) and multiple organ failure 1. In serious cases, clinical deterioration is often rapid, and in a large proportion the severe disease course is caused by systemic hyper-inflammation, the so called “cytokine storm” 2.

Cytokine storm is a condition of uncontrolled systemic hyper-inflammation caused by cytokine excess, leading to multi-organ failure and even death 3. The concept of cytokine storm was initially recognized in acute graft-versus-host disease in the process of hematopoietic stem cell transplantation 4. Researchers have since revealed that cytokine storm occurs in various diseases, such as malignancy, rheumatologic disease, and sepsis syndrome and is a concern in COVID-19 5. Even though a variety of treatments to mitigate cytokine storm have been introduced, no concrete treatment recommendations have been issued so far for COVID-19: in recent studies novel cytokine blockades targeting specific cytokines, such as interleukin (IL)-1 and IL-6 or Janus kinase (JAK) pathway have shown promising potential for the treatment of COVID-19. In addition, traditional anti-inflammatory drugs, including corticosteroids and colchicine, are also being explored for COVID-19 through active and vibrant researches 6.

In this study, we provide an overview of the cytokine storm caused by different etiologies and specific treatments thereof, focusing particularly on novel cytokine blockades. In addition, we discuss the pathophysiology and individual treatments for the cytokine storm in severe COVID-19. Last, we propose the potential of therapeutic plasma exchange (TPE) to treat cytokine storm in severe COVID-19. We believe that TPE is able to effectively remove inflammatory cytokines from the blood and can improve cytokine storm 7.

## Immunopathogenesis of cytokine storm

### Immune features of cytokine storm

Unpredicted adverse events in the phase 1 trial of the anti-CD28 monoclonal antibody TGN1412 provided clues about cytokine storm 8. In just a few hours after administration of the study medication, participants experienced serious complications from cytokine storm. The patients all commonly experienced sudden fever, respiratory and kidney failure, hypotensive shock, and diffuse coagulation disorders. Laboratory tests revealed anemia, neutrophilia, thrombocytopenia, and marked lymphopenia. These clinical features of multi-organ failure are believed to be caused by activation of the following four cascades: complement, kinin, clotting, and fibrinolysis systems 9. In addition, acute increases in various cytokines were also noted in the early phase: tumor necrosis factor-alpha (TNF-α) was observed within an hour, followed by IL-1 8.

Since then, through experience with various clinical diseases, including hemophagocytic lymphohistiocytosis (HLH), the concept that an excessive immune response can seriously damage the body and causes rapid clinical deterioration began to be established 10. Primary or familial HLH by genetic mutations and secondary HLH by specific viral infections, malignancies, autoimmune disorders, and iatrogenic causes are typical diseases accompanied by cytokine storm 11. In particular, cytokine storm caused by autoimmune disorders is historically called macrophage activation syndrome (MAS) 12, and cytokine release syndrome (CRS) refers to the cytokine storm that occurs after chimeric antigen receptor (CAR) T cell therapy 13. Sepsis is also an important cause for the development of cytokine storm, but it is not easy to discriminate cytokine storm from the clinical course of severe sepsis itself 14.

A variety of cytokines, including the IL-1 family, IL-6, IL-8, IL-10, TNF-α, and interferon (IFN)-γ, are involved in the development of a cytokine storm, although the key pathogenic cytokines appear to differ depending on the disease. IFN-γ is a key cytokine in primary HLH 15. IL-1ß plays a major role in systemic juvenile idiopathic arthritis (SJIA) 16, while in MAS associated with SJIA, IL-18 may be a key cytokine 17. Another representative cytokine, IL-6, is a key inflammatory molecule in CRS 18. Importantly, even if the clinical symptoms caused by a cytokine storm exhibit a common pattern, treatment must be individualized because the degree to which each cytokine contributes to the development of the disease can differ 3. On the other hand, cytokine storm in sepsis involves multiple factors, and thus, multi-directional suppression of inflammatory reactions is necessary.

Interestingly, leukocytopenia, particularly lymphopenia, has been found to be a typical finding in cytokine storm and to be associated with the severity thereof. One study has described a possible mechanism for lymphopenia in cytokine storm. After the TGN1412 trial, Muller et al. administered another anti-CD28 super-agonistic antibody, JJ316, to experimental rats and observed responses 19. Similar to those that occurred in the TGN1412 trial, including lymphopenia. Further investigations revealed that T cells were rapidly redistributed to secondary lymphoid organs, such as the spleen and lymph nodes, and were trapped with decreased mobility. Although we are not sure why these findings occur as a part of systemic hyper-inflammatory reactions, considering that lymphopenia was seen without infection in the trial, it is not likely that lymphopenia is induced directly by a pathogenic organism, such as a virus. Instead, we believe that lymphopenia might be the result of effective immune reactions against severe systemic infections for more efficient trafficking of immune cells to secondary lymphoid organs. One other notable thing from the TGN1412 trial is that the violent inflammatory responses were followed by reactive immunosuppression. The responses, called “second wave” or “immune paralysis,” were found to downregulate the previous inflammatory reactions, which is likely why TGN1412 was suspected as showing therapeutic effects on autoimmune disease in preclinical studies in rats 20.

Research has yet to reveal whether cytokine storm results from abnormal innate or adaptive immunity. As observed in the TGN1412 trial, rapid onset of cytokine storm after drug administration and a series of inflammatory responses following an acute increase of TNF-α suggests a problem with innate immunity 8. However, considering the elevation of soluble interleukin-2 receptor in secondary HLH 21 and the effects of standard treatment for HLH targeting dysregulated T cells 22, the problem of adaptive immunity is also thought to be the main mechanism of cytokine storm. Among various cytokines related to cytokine storm, IL-1 and IL-6 have been described as key cytokines in some patients. These cytokines are representative pro-inflammatory cytokines, along with TNF-α, that play an important role in acute phase responses of inflammation. Pattern recognition receptors (PRRs), such as toll-like receptor (TLR) and retinoic acid-inducible gene-1-like receptor (RLR), are proteins that present in the cytoplasm or cell membrane to recognize infection or tissue damage by combining with pathogen-associated molecular pattern (PAMP) derived from microorganisms or damage-associated molecular pattern (DAMP) resulting from tissue damage. IL-1, IL-6, and TNF-α are produced by dendritic cell and mononuclear macrophage that are activated by PRRs, and initiate a series of acute inflammatory reactions through activation of innate immunity 23. Meanwhile, however, IL-6, along with IFN-γ, is also important for T-cell mediated adaptive immunity 24. Therefore, dysregulated activation of T cells can be considered as a major pathological mechanism in a cytokine storm wherein IL-6 and IFN-γ are key cytokines. Taken together, although the clinical characteristics of cytokine storm are similar, underlying pathological mechanisms affecting the clinical course, prognosis, and treatment response in individual forms of cytokine storm can differ. Thus, it is important to find the key cytokine in various cytokine storms and to take a proper therapeutic approach by determining whether the instance is related with innate or adaptive immunity.

### Cytokine storm in severe COVID-19

Studies have indicated that the rapid clinical deterioration and high mortality risk in severe COVID-19 could be related to cytokine storm 6. One study showed that blood levels for various cytokines, such as IL-1ß, IFN-γ, IFN-γ-induced protein 10 (IP10), and monocyte chemoattractant protein 1 (MCP1), were elevated in COVID-19 25. In addition, patients admitted to the intensive care unit (ICU) had higher cytokine levels of IL-2, IL-7, IL-10, granulocyte colony-stimulating factor (G-CSF), IP10, MCP1, macrophage inflammatory protein 1- α, and TNF-α than those not requiring ICU treatment 25. Another study showed that IL-6 was more elevated in non-survivors than survivors from COVID-19, suggesting that COVID-19 mortality might be due to virus-activated cytokine storm 26.

**Figure 1:**
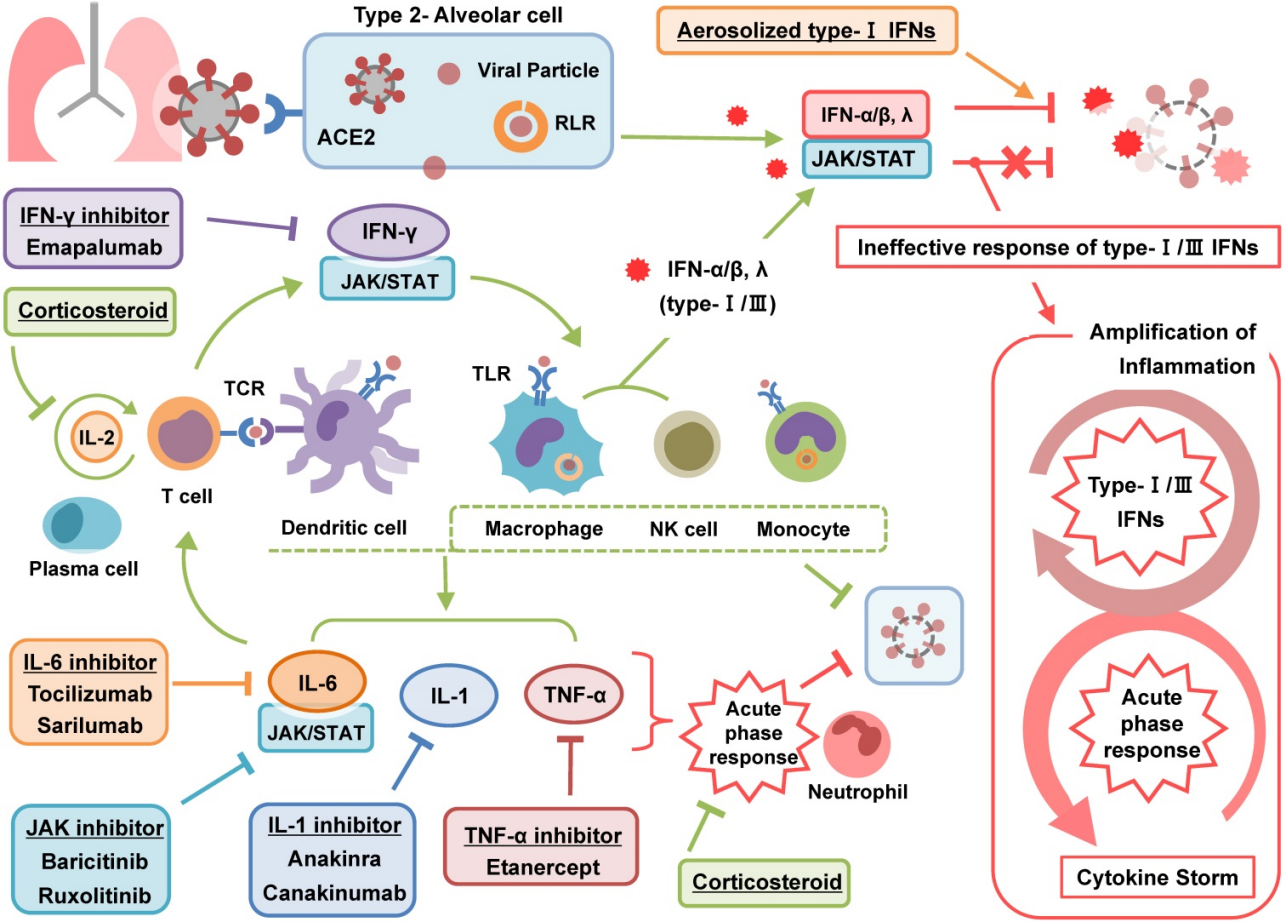
Pathophysiology and treatment of cytokine storm in COVID-19. Type-1 IFNs play a major role in inhibiting the early stage of COVID-19 infection. Dendritic cells and mononuclear macrophages that recognize viral antigens induce an acute phase response through pro-inflammatory cytokines such as IL-6, IL-1, and TNF-α. Among them, IL-6 stimulates T-cells to activate adaptive immunity. Activated T cells also stimulate macrophage and NK cells through IFN-γ to promote virus removal. Failure of the initial immune response by type-1 IFNs increases excessively the activity of immune system leading to cytokine storm. Aerosolized type-1 IFNs promotes an initial immune response to remove virus, corticosteroids and novel cytokine blockades inhibits immune responses to prevent cytokine storm. ACE2: angiotensin converting enzyme-2; COVID-19: coronavirus disease 2019; IFN: interferon; IL: interleukin; JAK/STAT; Janus kinase-signal transducer and activator of transcription; NK: natural killer; RLR: retinoic acid-inducible gene-1-like receptor; SARS-CoV-2: severe acute respiratory syndrome coronavirus 2; TCR: T cell receptor; TCZ: tocilizumab; TLR: toll-like receptor; TNF-α: tumor necrosis factor-alpha.

A recent article presented an interesting concept of a cytokine storm in COVID-19. The study argued that the cytokine storm in COVID-19 is the result from a failure of the immune system to remove the virus. They divided the cytokine storm into two stages 27: The first stage is a temporary immune-deficient condition that is similar to primary HLH. The subsequent secondary stage is an overactive immune state to compensate for the target clearance failure, which appears as a clinical manifestation of a cytokine storm. Another review article also described the cytokine storm associated with COVID-19 in a similar context 28. Cell and animal experiments investigating the effects of human coronavirus on cytokines have demonstrated delayed secretion of type Ⅰ and Ⅲ IFNs, including IFN α/ ß, in the early phase of infection and excessive secretion of pro-inflammatory cytokines from mononuclear macrophages in the later stage 29. Another study in which immune analysis was performed in COVID-19 patients highlighted profound impaired type 1 IFN responses characterized by a low level of IFN activity and downregulation of IFN stimulated genes. In addition, the study reported hyper-inflammatory responses represented by IL-6 and TNF-α 30. Taken together, these studies emphasize that a failure in initial type-Ⅰ and Ⅲ IFN responses to SARS-CoV-2 leads to an excessive late immune response and severe form of COVID-19 (**Figure 1**). In this respect we could figure out why severe COVID-19 is commonly accompanied by ARDS. The failure of immune response in initial period of SARS-CoV-2 infection induces generalized hyper-inflammation in lung that leads to acute lung injury and ARDS.

Many researchers believe that there are certain people who are vulnerable to cytokine storm in COVID-19, although clear evidence of this is lacking, at the moment. In support of this notion is a genetic predisposition to cytokine storm in primary HLH, which is already well documented. Primary HLH results from genetic defects in a perforin and granzyme dependent pathway in the action of natural killer (NK) cells and cytotoxic T lymphocytes 31. Additionally, a few studies have also indicated genetic predispositions in other cytokine storm diseases. One study showed that some patients with SJIA carried perforin gene mutations related to reduce perforin activity 32. Another study demonstrated that perforin expression was also reduced in patients with SJIA and could be restored following clinical improvement after stem cell transplantation 33. Accordingly, we suspect there could be a genetic predisposition that makes some people more vulnerable than others to cytokine storm, leading to severe COVID-19 34, 35. To confirm this, more epidemiological studies on patients presenting with cytokine storm related to COVID-19 are needed in the future.

## Treatment of cytokine storm

### Treatments with specific cytokine inhibitors

Current recommendations for the treatment of primary and secondary HLH are based on specific treatment protocols 22. The mainstay of treatment is the use of corticosteroids, cyclosporine and etoposide, which are commonly used to target dysregulated T cells. The range of effects of these drugs is extensive and inhibits various mechanisms in the pathogenesis of HLH. Specific cytokine blockades have also proven successful in treating various types of HLH, although not as a general therapeutic option: it appears to only be effective when a target cytokine plays a key role in the disease.

During the COVID-19 pandemic, many therapeutic drugs and interventions have been attempted to treat severe COVID-19 (**Table 1**). In light of successes in the treatment of various types of cytokine storm, a few cytokine blockades have been explored in the treatment of severe COVID-19 36.

### Inhibition of IL-1 signaling

IL-1 is one of the major pro-inflammatory cytokines. It is comprised of two types of ligands, IL-1α and IL-1ß, of which IL-1ß plays a major role with systemic effects. IL-1 is primarily produced by innate immune cells, such as macrophages and monocytes. IL-1 exerts pro-inflammatory actions to recruit immune cells and to induce secondary cytokine production resulting in acute phase reactions (**Figure 1**). Some researchers argue that diseases caused by dysregulated IL-1 ought to be considered as auto-inflammatory diseases, not auto-immune diseases, because IL-1 is not directly involved in adaptive immunity, as with T and B cells 37.

Anakinra, a recombinant IL-1 receptor antagonist, has been effectively used to treat auto-inflammatory diseases, from rheumatoid arthritis to cytokine storm 38. Another IL-1 inhibitor, canakinumab which is a human monoclonal antibody neutralizing IL-1ß, has also been found to be useful in treating various types of rheumatologic diseases, with the advantage of prolonged action 39. Inhibition of IL-1 naturally exists as a feedback mechanism of self-regulation. Anakinra is a modified form of the human IL-1 inhibitor. In that context, the action of anakinra is expected to be safe because it is similar to its physiologic mechanism. Despite the infection risk, it is remarkably safe, relative to other agents in the same line. Furthermore, the effects of anakinra are not limited to the treatment of rheumatologic diseases. A re-analysis of a randomized controlled trial (RCT) demonstrated that anakinra reduces mortality in sepsis patients experiencing cytokine storm 40.

A few studies have suggested beneficial effects with the use of anakinra in COVID-19. A recent retrospective cohort study of patients with COVID-19 and ARDS showed that high-dose anakinra could be used safely and improved respiratory function 41. Another prospective cohort study of patients with severe COVID-19 pneumonia demonstrated that anakinra reduced the need for mechanical ventilation and mortality without serious side-effects 42. Although both studies showed promising results for anakinra on severe COVID-19, further validation is needed through RCTs.

### Inhibition of IL-6 signaling

IL-6 is an important pro-inflammatory cytokine that has pleiotropic effects. It is induced by infection or tissue injury and rapidly elicits acute reactions to minimize them. IL-6 promotes the production of various acute phase proteins in hepatocytes and induces the differentiation of immune cells, such as B and T cells (**Figure 1**). In addition, IL-6 is involved in the metabolism of iron by regulating hepcidin to make a microenvironment prohibitive against infection. Taken together, IL-6 plays a role in priming inflammatory responses and in activating adaptive immunity against infection or injury 24.

A few IL-6 inhibitors have been used in auto-immune disorders and related cytokine storm. Tocilizumab (TCZ), an anti-IL-6 receptor monoclonal antibody, has been used to treat various types of rheumatologic diseases, including rheumatoid arthritis, with excellent efficacy. Furthermore, it has been approved by the FDA in the management of CRS after CAR T-cell therapy 43. On the other hand, it has not been found to show any effects against sepsis syndrome.

Many studies have demonstrated that IL-6 is significantly elevated in patients with COVID-19. One study showed that IL-6 levels in COVID-19 patients were very high and comparable to those in CAR T cell CRS 44, 45. Moreover, studies have suggested that IL-6 is predictive of poor outcomes in COVID-19. One study showed that non-survivors had higher IL-6 levels than survivors 46, and another recent study suggested that a high level of IL-6 predicted the risk of requiring mechanical ventilation 47. A recent prospective cohort study indicated that high levels of IL-6 and d-dimer reflected systemic inflammation and thrombotic condition, and predicted in-hospital mortality of COVID-19 48.

Two IL-6 inhibitors are being attempted in treatment of COVID-19. In a few case series, sarilumab and tocilizumab have shown beneficial effects in reducing severity and mortality in severe COVID-19. A recent retrospective cohort study of 1,351 patients with COVID-19 and pneumonia showed that tocilizumab significantly reduced the mechanical ventilation risk or death 49. In an observational study of 154 patients requiring mechanical ventilation, tocilizumab reduced the risk of death by 45%. However, this study also showed that, with tocilizumab, the risk of superinfection significantly increased 50. Although RCTs are required to validate these clinical effects, the outlook for these drugs in the treatment of COVID-19 seems encouraging.

### Inhibition of TNF-α signaling

TNF-α is a cytokine primarily produced by activated macrophages in the acute inflammatory phase (**Figure 1**). In the TGN1412 trial, TNF-α was first to be elevated among several elevated cytokines 8. Considering the role of TNF-α in the inflammatory response, TNF-α blockade is expected to have promising effects in various types of cytokine storm. However, in contrast to treatment of rheumatoid arthritis of rheumatoid diseases, its effectiveness has not been proven in the actual treatment of cytokine storm. While a few case reports have described the usefulness of TNF-α blockade with etanercept in MAS 51, 52, there are no relevant studies proving the effects systemically, and two studies failed to document beneficial effects for TNF-α blockade in sepsis syndrome 53, 54.

Anti-TNF-α therapy has not been actively attempted in COVID-19. Reports of COVID-19 infection in patients who maintain anti-TNF-α therapy against existing inflammatory bowel disease have suggested that anti-TNF-α therapy maintenance is safe; however, clear evidence of clinical improvement therewith is lacking 55. A recent interesting study showed that TNF-α mediated acute lung injury was reduced by using an aptamer targeting TNF-α. Considering that acute lung injury is a characteristic finding in COVID-19, this study provides a possible therapeutic approach to treating COVID-19 cytokine storm accompanied by lung injury 56.

### Inhibition of IFN-γ signaling

IFN-γ is secreted by several immune cells, including macrophages, NK cells, and T cells, and plays a role in stimulating the main inflammatory effector cells directly (**Figure 1**). Thus, IFN-γ is considered a major effector cytokine in various cytokine storm disorders. In particular, IFN-γ is a key cytokine in primary HLH, which is caused by hyperactivity of the immune system in response to a failure to eliminate pathogens due to perforin defects. In HLH, IFN-γ is excessively stimulated and leads to a cytokine storm. Emapalumab, an anti- IFN-γ monoclonal antibody, has been used to treat primary HLH since it was approved by the FDA in 2018 57. Several studies have documented clinical effects for emapalumab by confirming decreases in blood levels of C-X-C motif chemokine ligand 9 (CXCL9), an IFN-γ derived chemokine that is a good indicator to measure IFN-γ activity after use of emapalumab. A recent interventional study indicated that emapalumab had clinical effects without specific side effects in primary HLH treatment 58. Another study described clinical effects for emapalumab in refractory HLH caused by Epstein-Barr virus (EBV) infection 59. Interestingly, the study indicated that excess IFN-γ contributed to immune paralysis and that emapalumab could reduce cytokine storm and effectively clear persistent EBV by reversing immune paralysis. For treating COVID-19, there are no significant trials supporting the use of IFN-γ inhibitors yet. Instead, many researchers have attempted to administer type 1 IFNs, such as IFN α, ß and κ, in the early phase of COVID-19 60, 61, as recent studies have suggested that decreased type Ⅰ and Ⅲ IFNs response to SARS-CoV-2 plays an important role in the development of the observed cytokine storm associated with COVID-19 62.

### Inhibition of JAK pathway

JAK is an intracellular tyrosine kinase that mediates signals from cytokines, hormones, and growth factors. The Janus kinase-signal transducer and activator of transcription (JAK/STAT) pathway is commonly involved in various cytokine activation processes (**Figure 1**). JAK inhibitors, including ruxolitinib and baricitinib, have been used to treat a variety of autoimmune and hematology diseases. JAK inhibition is thought to be able to effectively suppress cytokine storm because it can non-selectively inhibit the activity of many cytokines. On the other hand, non-selective inhibition of the immune response poses a risk of secondary infection, because it also suppresses the innate immune system that fights against pathogenic microorganisms, such as viruses, bacteria, or fungi. Moreover, because the JAK/STAT pathway is involved in several physiological mechanisms other than the immune response, inhibition of these pathways can potentially lead to unexpected side effects.

JAK inhibition in COVID-19 appears to offer two clinical advantages. JAK inhibitors block cytokine signaling, thereby reducing excessive inflammatory responses, as well as the entry of SARS-CoV-2, in the early phase of infection 63. As is well known, SARS-CoV-2 enters the body through angiotensin converting enzyme-2 (ACE2) on alveolar type 2 cells in the lungs, and several regulators are involved in mediating endocytosis and intracellular transport through ACE2. AP2-associated protein kinase-1, one such regulator, is also a target of JAK inhibitors, particularly baricitinib. Thus, JAK inhibitors can impede the entry and proliferation of SARS-CoV-2 64. Accordingly, several JAK inhibitors, including baricitinib, ruxolitinib, and fedratinib, are being studied in the treatment of severe COVID-19. A recent multi-centered RCT of ruxolitinib in patients with COVID-19 noted faster clinical improvement with the drug, although the results lacked statistical significance 65. Another multi-centered retrospective study demonstrated that baricitinib reduced the rate of ICU admission and fatality and increased discharge rates 66.

### Other anti-inflammatory or immunosuppressive agents

In addition to cytokine blockade, various anti-inflammatory therapies are being applied in an attempt to treat COVID-19. This trends show that anti-inflammatory treatment is as important as anti-viral treatment in the treatment of COVID-19. As with any serious illness, many clinicians are currently using glucocorticoids as an empirical treatment for severe COVID-19. Unlike specific cytokine inhibitors, glucocorticoids are believed to be effective against cytokine storm by inhibiting multiple inflammatory targets (**Figure 1**). Glucocorticoids not only have excellent immunosuppressive effects on immune cells, but also have anti-inflammatory effects by inhibiting the production of major inflammatory molecules, including prostaglandins and leukotrienes. Evidence supporting the use of glucocorticoids in severe COVID-19, however, is lacking 67, 68. Although this matter is still argued, several studies have positively reported steroid effects in severe COVID-19 69, 70.

Colchicine is an anti-inflammatory drug that is commonly used in gouty arthritis. The main action of colchicine is to impede the function of neutrophils, and it has the effect of inhibiting IL-1ß activity by inhibition of the inflammasome complex. A few studies are investigating the clinical effects of colchicine on COVID-19 71. A recent RCT demonstrated that colchicine delays the time to clinical deterioration in COVID-19 72.

Some clinicians have attempted to use stem cells for the treatment of severe COVID-19 73. Although the effectiveness of stem cell therapy in existing immune diseases has not been clearly demonstrated, researchers have argued that stem cell therapy has immunomodulatory effects and helps to differentiate immune cells.

Interestingly, a few researchers have proposed the use of low-dose radiation therapy to control hyper-inflammatory states in severe COVID-19 74, 75. According to the cited studies, low-dose radiation (usually < 1.0 Gy) has immune-modulating effects on immune cells, changing them into an anti-inflammatory phenotype. Thus, in the patients with COVID-19 and ARDS, low-dose radiation on both lungs could potentially facilitate clinical improvements in hyper-inflammatory lung injury.

Several drugs have been experimentally shown to be effective in reducing cytokine storm associated with influenza infection, which is similar to COVID-19. Peroxisome proliferator-activated receptors (PPARs) agonists, cyclooxygenase (COX) inhibitors 76, and sphingosine-1-phosphate receptor 1 (S1P1) 77 agonists have been shown in several experimental studies to be effective in hindering cytokine storm in severe influenza infections. Although S1P1 agonist is known to induce lymphopenia, one study suggested that it might be helpful to mitigate the cytokine storm induced ARDS by preventing trafficking of immune cells to the lungs 78. A recent review described the use of nanomedicine to modulate macrophage dysfunction in cytokine storm. According to the researches, dysfunctional macrophage is critical in the pathogenesis of cytokine storm, and thus selectively inhibiting dysregulated macrophages using liposomes or synthetic nanoparticles can be a novel therapeutic approach for the treatment of cytokine storm 79. However, the use of these drugs in these instances is not supported by evidence in human subjects and clinical trials, and requires substantial clinical data.

### Issues with treatment of cytokine storm associated with COVID-19

Several studies have shown that cytokine plasma concentrations exhibit diurnal variations. According to a meta-analysis study, IL-6 concentrations are lowest in the morning and peak twice during the time from afternoon to morning 80. Another study showed that several cytokines, including IL-6 and IL-1, had higher concentrations in the afternoon than in the morning 81. Both studies commonly proposed diurnal variations in cortisol as a contributing factor to the diurnal variations in the cytokines. It also seems that they may also be affected by different physiological mechanisms that have not yet been identified. Therefore, diurnal variations in these cytokines should be considered in the diagnosis and treatment of cytokine storm and regarded as an important factor when determining the appropriate timing of drug administration in cytokine-targeted therapy.

The cytokine storm caused by COVID-19 appears to be more similar to that in sepsis syndrome than that in HLH in hematologic or rheumatologic diseases. The cytokine storm related to COVID-19 was caused by infection as in the case of sepsis syndrome. As the cytokine storm caused by infections is likely involving more complex mechanisms than that by non-infectious causes, the effect of targeted treatment on a specific cytokine may be limited. Nonetheless, the results of ongoing research do not seem to be discouraging, and additional verification through RCTs is required.

Treatment of COVID-19 is largely divided into two parts: antiviral treatment to inhibit SARS-CoV-2 replication and anti-inflammatory treatment to reduce systemic inflammation. Treatment of the cytokine storm belongs to the latter, and new targeted therapies for cytokines and corticosteroids have been attempted. Anti-inflammatory therapy in severe COVID-19 poses one critical issue. Anti-inflammatory treatment is aimed at clinical improvement through suppression of excessive immune responses; while on the other hand, the immunosuppressive effect of anti-inflammatory treatment paradoxically decreases the clearance of the virus from the body and increases the risk of secondary bacterial infections. In influenza infections, despite no definitive conclusion on their effects and risks, steroids are considered empirical drugs in severe cases. During influenza pandemic periods, many clinicians use steroids to treat serious clinical complications, including ARDS 82. To compensate for decreased capacity of viral eradication and to avoid the risk of secondary bacterial infections, simultaneous administration of anti-viral agents (e.g., oseltamivir) and broad-spectrum antibiotics are essential in severe influenza infection 83. On the contrary, the anti-inflammatory treatment for severe COVID-19 is in a very disadvantageous position, because there is no proven effective anti-viral agent at the moment. Although an observational study and a preliminary report from an RCT on the effectiveness of the use of remdesivir in severe COVID-19 have recently been released, further verification through complete and reliable RCTs is required 84, 85. In COVID-19, immunosuppressive agents, including cytokine blockades and steroids, should be used appropriately only when an excessive immune response is evident, and thus, a proper clinical diagnosis of cytokine storm is essential.

Early detection of cytokine storm and immediate initiation of treatments to reduce severity are essential for the treatment of severe COVID-19. A recent report focused on the rheumatologist's role in COVID-19 era, providing guidelines for the early diagnosis of cytokine storm, and suggested the need for cooperation with rheumatologists as professionals with experience in treating cytokine storm and in using various immunosuppressants 86. Meanwhile, many studies have emphasized the importance of hyperferritinemia in early diagnosis of cytokine storm 6. Serum ferritin can be easily measured in clinical laboratories and reflects inflammatory states well. Despite the lack of cut-off values, a significant elevation in ferritin and typical clinical features are suggestive of cytokine storm in COVID-19 86.

### Cytokine removal by blood purification

The efficacies of various immunosuppressive measures on cytokine storm are under investigation. While we are eagerly awaiting such results, other therapeutic measures that are able to reduce cytokine levels within hours of application are readily available. Direct removal of such pro-inflammatory stimuli might reduce the likelihood of progressive organ damage 87, a hallmark of COVID-19.

Many studies have investigated the removal of inflammatory cytokines through blood purification 88. Continuous renal replacement therapy (CRRT), as a type of hemofiltration or hemodiafiltration method, has shown clinical benefits from removing inflammatory molecules, beyond just replacing impaired kidney function. Previous studies have demonstrated that high doses of hemofiltration afforded better outcomes than standard doses in critical patients. These results suggest that high clearance of inflammatory cytokines might exert clinical benefits 89. Meanwhile, a systemic review investigating the effects of extracorporeal cytokine removal has indicated that high cut-off techniques remove cytokines more effectively through the larger pores of the membrane 90.

### Application of therapeutic plasma exchange in the treatment of cytokine storm

TPE is a more specific method to remove molecules more effectively from plasma than CRRT. It is usually used to treat antibody-mediated severe diseases, such as thrombotic microangiopathies, glomerulonephritis forms, Guillain-Barré syndrome, and others 91. Although TPE is not accepted as a standard treatment for HLH, a few studies have suggested a promising role for TPE in primary and secondary HLH. One study showed that inflammatory states were reduced after TPE therapy in children with hyperferritinemia and secondary HLH 92, and another case study reported that TPE facilitated a rapid improvement in patients with hemophagocytic syndrome during HLH-2004 protocol based standard therapy 93. A recent case series showed that early use of TPE along with immunosuppressive therapy was an effective treatment strategy for steroid refractory MAS-HLH 94.

TPE has also been used as an alternative treatment for severe sepsis (**Table 2**). Nevertheless, there is no clear evidence to recommend the use of TPE in severe sepsis yet, because the available trials have shown conflicting results 95, 96. Still, a few studies support the role of TPE in sepsis. A well-conducted systemic review indicated that TPE could reduce mortality in adult patients with sepsis 97. A recent non-randomized prospective study demonstrated that early TPE was associated with a rapid reduction of norepinephrine in septic shock, maintaining mean arterial pressure above 65 mmHg. Furthermore, it could also reduce pro-inflammatory cytokines (IL-6, IL-1ß, and angiopoietin-2) 98. Another investigation involving subjects with septic shock demonstrated that TPE partially reversed coagulation disorders in patients requiring high doses of norepinephrine 99.

In regard to TPE use in cytokine storm, TPE has several strengths. First, TPE can elicit immediately clinical effects in rapidly deteriorating cytokine storm. Early use of TPE may compensate for relatively slow action of immunosuppressants. Second, TPE exhibits non-selective properties in the elimination of inflammatory cytokines. It is believed that non-selective removal of cytokines is particularly suitable for the treatment of sepsis-induced cytokine syndrome 100, because the occurrence of a cytokine storm related to sepsis is believed to be the result of a complex mechanism involving various inflammatory factors 101. In that context, we prefer centrifugal-type TPE over filtration-type, because centrifugal TPE is less selective in separating plasma, although we still believe that TPE itself could provide clinical benefits regardless of the type. A previous study in the patients with Guillain-Barré syndrome also demonstrated that centrifugal TPE had shorter time to onset and greater clinical effects than double filtration plasmapheresis 102. Last, bulk removal of cytokines by TPE could obviate negative effects on immune cells and improve the function of monocytes and/or macrophages, thereby reversing immune paralysis. Overall, the immunomodulation effects by TPE could reinforce the immunity against COVID-19 and provide the chance of overcoming cytokine storm by increasing susceptibility to simultaneously administered immunosuppressants 100.

### Proposal for the use of therapeutic plasma exchange in COVID-19

Influenza infection as a viral disease is more similar to COVID-19 than bacterial sepsis. Severe COVID-19 has clinical features similar to severe influenza infection, characterized by acute lung injury and multiple organ failure. A few case studies have reported that TPE could be helpful in serious influenza infections. One case study showed that three children with severe influenza infections were able to recover from acute respiratory failure and hemodynamic shock through a rescue therapy comprising three consecutive sessions of TPE 103. Another case study showed that three patients with influenza-associated encephalopathy had markedly improved with two to three sessions of TPE every other day in combination with methylprednisolone injections (30 mg/kg). In addition, the study indicated that a key pro-inflammatory cytokine, IL-6, was significantly reduced in blood and cerebrospinal fluid after repetitive TPE 104.

Currently, remdesivir is being considered as a potential drug for treating COVID-19; however, in severe COVID-19, both antiviral treatment that suppresses the activity of the virus and anti-inflammatory treatment that can reduce excessive inflammation in the body are essential. In our eyes, rescue therapy comprising TPE as an alternative treatment early in serious cases with signs of a rapid worsening disease course and features of cytokine storm might be warranted. Despite the lack of a solid basis for the availability of TPE, even in severe infectious conditions such as sepsis, we believe that practical experiences with TPE use in various clinical diseases provide clues on TPE availability. Furthermore, the simple and reasonable hypothesis that, by exchanging plasma, cytokines and viruses can be effectively eliminated is worth considering. In this regard, a recent case report described the effects of TPE on COVID-19 105. In that case report, a patient with severe respiratory failure and anti-phospholipid syndrome by COVID-19 was given three sessions of plasma exchange. After plasma exchange, the patient showed clinical improvements with reduced titers of antiphospholipid antibodies and inflammatory markers, including IL-6. Another case series demonstrated that TPE had effects on treating COVID-19-related autoimmune meningoencephalitis 106. In that report, ferritin levels reflective of hyper-inflammatory states were reduced significantly after repetitive plasma exchange. Other than that, a few case studies of COVID-19 patients with ARDS and shock have indicated that TPE dramatically improved clinical conditions and inflammatory markers, even after small numbers of trials 107-110. A recent observational cohort study also showed that TPE afforded clinical benefits of a higher extubation rate and lower mortality rate at 28-day in COVID-19 patients with respiratory failure than non-TPE cases 111. Currently, while there are increasing case reports indicating the usefulness of TPE use in severe COVID-19 (**Table 3**) and while many clinicians might be using it in clinical practice, RCTs are still required to attain relevant clinical evidence in support thereof 112.

There are several clinical issues with the use of TPE in COVID-19. First, it poses risks of bleeding and catheter infection. In addition, it can cause electrolyte imbalances, such as hypocalcemia and hypokalemia, depending on the type of anti-coagulating agents or replacement fluids. TPE can also occasionally induce unpredicted anaphylactic shock due to the use of blood materials. However, the incidences of these complications are thought to be relatively small, and most cases can be controlled with medical support. Although TPE can be applied without technical difficulties in experienced centers, active involvement of medical staff in this procedure and capacity issues (most centers are limited with machines) only allow for the use of TPE in selected patients. Above all, the primary critical issue with TPE in COVID-19 is that the use of non-convalescent plasma as a substitution fluid could reduce protective antibodies against SARS-CoV-2. According to the clinical experiences of a few researchers, SARS-CoV-2-specific IgG and IgA antibodies were detected in waste bags, and circulating antibodies were also reduced during TPE application 113. However, despite these concerns, a few studies have demonstrated the clinical benefits of TPE in severe COVID-19, and we believe that timely, appropriate application of TPE in selected cases presenting with cytokine storm could be beneficial.

Since clinical deterioration of COVID-19 is mostly accompanied by worsening pneumonia and development of ARDS, we recommend initiating TPE when there are signs of respiratory failure requiring mechanical ventilation. In particular, highly elevated ferritin levels in blood, and the estimation of myocardial injury using high-sensitivity cardiac troponin I may also be considered useful 114, 115.

Severe COVID-19 is usually accompanied by hypotensive shock, which makes it difficult to maintain extracorporeal circulation. Therefore, it is better to start TPE early when blood pressure is maintained. In addition, referring to existing reports, we believe that TPE may be appropriate for use every day, or every other day. Previous reports have generally applied TPE two to three times in total; however, we believe that TPE can continue as long as there is no particular complication and exchangeable plasma is supplied smoothly. In addition, discontinuation may be considered if critical clinical conditions can be controlled by therapeutic methods other than TPE. In a few studies, two to 14 daily sessions, on average, were performed, with close monitoring of disseminated intravascular coagulation markers 116, 117. A target calculated plasma volume exceeding 1 should be considered, and fresh frozen plasma is recommended to be used as a substitution fluid, due to the replenishment of anti-infective factors in serum. Ideally, convalescent plasma, if available, should be used, even if only at the end of a session 118-120. Regarding existing TPE techniques, the centrifugal method might be preferable, as RCTs on TPE in septic shock have showed positive results with using centrifugation, although filtration types are also considered useful. Also, precise fluid overload control is necessary as decreasing vessel permeability is positive but can further impair pulmonary saturation.

## Conclusion

Cytokine storm is a severe clinical condition caused by systemic hyper-inflammation, resulting in severe injury to multiple organs, including the lungs, and even death. In that sense, anti-inflammatory treatment, along with anti-viral therapy, remains an important part of treatment for COVID-19. Currently, many anti-inflammatory agents have been attempted to treat severe COVID-19, and a large number of mixed results are being reported. With mounting evidence, we believe novel cytokine inhibitors, including tocilizumab, anakinra, and baricitinib, are promising options for treating severe COVID-19.

In this review, we described TPE as a realistic alternative in the treatment of cytokine storm associated with COVID-19. We believe that TPE can have clinical benefits on severe COVID-19 when initiated promptly after early diagnosis based on rapid clinical deterioration and high inflammatory parameters, such as serum ferritin and high-sensitivity cardiac troponin I. We strongly recommend using TPE with convalescent plasma. Overall, we propose that TPE can be useful in combination with other potentially effective options for treating severe COVID-19.

## Contributions

All authors made substantial contributions to all of the following: (1) conception and design of the study, data acquisition, or analysis and interpretation of data; (2) drafting or critical revision of the article for intellectual content; and (3) approval of the final version of the submitted manuscript.

## Figures and Tables

**Table 1:** Therapeutic options for the treatment of cytokine storm in severe COVID-19

Targeted inhibition	Drugs or Interventions	Previous established or alternative indications
IL-1	Anakinra, Canakinumab	RA, MAS-HLH
IL-6	Tocilizumab, Sarilumab, Siltuximab	RA, SJIA, CRS, Castleman disease
TNF-α	Etanercept	RA, SJIA
IFN-γ	Emapalumab	Primary HLH
JAK	Baricitinib, Ruxolitinib	RA, MF, PV
Non-selective	Glucocorticoid	Various autoimmune diseases and hematologic malignancies
Non-selective	Colchicine	Gout
Non-selective	Mesenchymal stem cell	Not yet documented, Regenerative medicine
Non-selective	Plasma exchange	Various Ab-mediated diseases, Hyperviscosity syndrome
Non-selective	Intravenous immunoglobulin	Various autoimmune and infectious diseases, Ab deficiency disorders
Non-selective	Convalescent plasma	Not yet documented, Rescue therapy in severe infectious diseases
Non-selective	Radiation	Tumor

Ab: antibody; COVID-19: coronavirus disease 2019; CRS: cytokine release syndrome; HLH: hemophagocytic lymphohistiocytosis; IFN: interferon; IL: interleukin; JAK: Janus kinase; MAS: macrophage activation syndrome; MF: myelofibrosis; PV: polycythemia vera; RA: rheumatoid arthritis; SJIA: systemic juvenile idiopathic arthritis; TNF-α: tumor necrosis factor-alpha.

**Table 2:** Studies on the effects of therapeutic plasma exchange in severe sepsis or septic shock

Authors (year)	Type of Study	Subjects	Clinical outcomes
Reeves et al. (1999)	Multicenter, Prospective, RCT	Adults (n, 22); Children (n, 8) with sepsis	No significant difference in mortality at 14-day; No significant reduction in the risk of death in TPE; No effects on IL-6, G-CSF, and thromboxane-B.
Busund et al. (2002)	Single-center, Prospective, RCT	Adults (n, 106) with septic shock	Mortality at 28-day: TPE vs. Control; 33·3 vs. 53·8 (%) (*p*=0·050); Absolute risk reduction: 20·5%.
Nguyen et al. (2008)	Single-center, Prospective, RCT	Children (n, 10) with Thrombocytopenia and MOF	Improved ADAMTS-13 activity and organ function; at median 12-day in TPE group (*p*<0·05).
Rimmer et al. (2014)	Systemic review Meta-analysis	Four RCTs including children and adults	No significant reduction of all-cause mortality in overall patients; But, significant reduction of all-cause mortality in adult patients; (Risk Ratio 0·63, CI 0·42-0·96)

CI: confidence interval; G-CSF: granulocyte colony-stimulating factor; IL: interleukin; JAK: Janus kinase; MOF: multiple organ failure; RCT: randomized controlled trial; TPE: therapeutic plasma exchange.

**Table 3:** Case studies on the effects of therapeutic plasma exchange in COVID-19

Authors	Subjects	Prescription of TPE ^a^	Clinical outcomes
Keith et al.	One patient with pneumonia, shock, and multi-organ failure	1/ 4.5L/ FFP	Improved respiratory condition and hypotensive shock, increased heart function
Shi et al.	One patient with ARDS, shock	3/ 6L/ FFP	Improved respiratory condition and hypotensive shock
Morath et al.	Five patients with respiratory failure	1~2/ 3.39L/ FFP	Improved respiratory condition and hypotensive shock, Decrease in inflammatory marker (IL6, ferritin, D-dimer)
Adeli et al.	Eight patients with ARDS and shock	3~5/ 2L/ FFP, albumin	Improved respiratory condition
Dogan et al.	Six patients with COVID-19-related autoimmune meningoencephalitis	1~3/ no data/ albumin	Clinical improvement including meningoencephalitis, Decrease in serum ferritin
Khamis et al.	Eleven patients with ARDS or pneumonia	5/ one times body plasma volume/ FFP	Higher extubation rate, lower mortality at 14 and 28 days compared to non-TPE cases

^a^ Prescription of TPE is presented as total number of trials/ dose per session/ type of substitution fluid. ARDS: acute respiratory distress syndrome; COVID-19: coronavirus disease 2019; FFP: fresh frozen plasma; IL: interleukin; TPE: therapeutic plasma exchange.

## Abbreviations

Ab: antibody
ACE2: angiotensin converting enzyme 2
ARDS: acute respiratory distress syndrome
CAR: chimeric antigen receptor
COVID-19: coronavirus disease 2019
COX: cyclooxygenase
CRRT: continuous renal replacement therapy
CRS: cytokine release syndrome
CXCL9: C-X-C motif chemokine ligand 9
DAMP: damage-associated molecular pattern
EBV: Epstein-Barr virus
FFP: fresh frozen plasma
G-CSF: granulocyte colony-stimulating factor
HLH: hemophagocytic lymphohistiocytosis
ICU: intensive care unit
IFN: interferon
IL: interleukin
IP10: IFN-γ-induced protein 10
JAK: Janus kinase
JAK/STAT: Janus kinase/signal transducers and activators of transcription
MAS: macrophage activation syndrome
MCP1: monocyte chemoattractant protein 1
MF: myelofibrosis
MOF: multiple organ failure
NK: natural killer
PAMP: pathogen-associated molecular pattern
PPARs: Peroxisome proliferator-activated receptors
PRRs: pattern recognition receptors
PV: polycythemia vera
RA: rheumatoid arthritis
RCT: randomized controlled trial
RLR: retinoic acid-inducible gene-1-like receptor
S1P1: sphingosine-1-phosphate receptor 1
SARS-CoV-2: severe acute respiratory syndrome coronavirus 2
SJIA: systemic juvenile idiopathic arthritis
TCR: T cell receptor
TCZ: tocilizumab
TLR: toll-like receptor
TNF-α: tumor necrosis factor-alpha
TPE: therapeutic plasma exchange
